# The cutaneous beta human papillomavirus type 8 E6 protein induces CCL2 through the CEBPα/miR-203/p63 pathway to support an inflammatory microenvironment in epidermodysplasia verruciformis skin lesions

**DOI:** 10.3389/fcimb.2024.1336492

**Published:** 2024-03-06

**Authors:** Luca Vella, Anna Sternjakob, Stefan Lohse, Alina Fingerle, Tanya Sperling, Claudia Wickenhauser, Michael Stöckle, Thomas Vogt, Klaus Roemer, Monika Ołdak, Sigrun Smola

**Affiliations:** ^1^Institute of Virology, Saarland University Medical Center, Homburg, Germany; ^2^Institute of Virology, University of Cologne, Cologne, Germany; ^3^Institute of Pathology, University of Cologne, Cologne, Germany; ^4^Department of Urology and Pediatric Urology, Saarland University Medical Center, Homburg, Germany; ^5^Department of Dermatology, Saarland University Medical Center, Homburg, Germany; ^6^Jose Carreras Center for Immune and Gene Therapy, Saarland University Medical Center, Homburg, Germany; ^7^Department of Histology and Embryology, Medical University of Warsaw, Warsaw, Poland; ^8^Helmholtz Institute for Pharmaceutical Research Saarland (HIPS), Helmholtz Centre for Infection Research, Saarbrücken, Germany

**Keywords:** HPV, E6, inflammation, CCL2, macrophage, p63, C/EBP, epidermodysplasia verruciformis

## Abstract

Human papillomavirus type 8 (HPV8), a cutaneous genus beta HPV type, has co-carcinogenic potential at sun-exposed sites in patients suffering from the inherited skin disease epidermodysplasia verruciformis (EV). We had previously shown that Langerhans cells responsible for epithelial immunosurveillance were strongly reduced at infected sites and that the HPV8 E7 protein interferes with the CCAAT/enhancer-binding protein (C/EBP)β to suppress the Langerhans cell chemokine CCL20. At the same time, however, we observed that EV lesions are heavily infiltrated with inflammatory immune cells, which is similar to the situation in HPV8 E6 transgenic mice. To identify critical inflammatory factors, we used a broad multiplex approach and found that the monocyte attracting chemokine CCL2 was significantly and strongly induced by HPV8 E6 but not E7-expressing HaCaT cells, which were used as a model for UV-damaged skin keratinocytes. Conditioned media from HPV8 E6-expressing keratinocytes enhanced CCL2-receptor (CCR2)-dependent monocyte recruitment *in vitro*, and macrophages predominated in the stroma but were also detected in the epidermal compartment of EV lesions *in vivo*. CCL2 induction by HPV8 E6 was even stronger than stimulation with the proinflammatory cytokine TNF-α, and both HPV8 E6 and TNF-α resulted in substantial suppression of the transcription factor C/EBPα. Using RNAi-mediated knockdown and overexpression approaches, we demonstrated a mechanistic role of the recently identified C/EBPα/miR-203/p63 pathway for HPV8 E6-mediated CCL2 induction at protein and transcriptional levels. Epithelial co-expression of p63 and CCL2 was confirmed in HPV8 E6-expressing organotypic air–liquid interface cultures and in lesional EV epidermis *in vivo*. In summary, our data demonstrate that HPV8 oncoproteins actively deregulate epidermal immune homeostasis through modulation of C/EBP factor-dependent pathways. While HPV8 E7 suppresses immunosurveillance required for viral persistence, the present study provides evidence that E6 involves the stemness-promoting factor p63 to support an inflammatory microenvironment that may fuel carcinogenesis in EV lesions.

## 1 Introduction

Human papillomaviruses (HPVs) are epitheliotropic double-stranded DNA viruses that cause hyperproliferative lesions in mucosa and skin. More than 200 HPV types have been phylogenetically classified into five different genera α, β, γ, δ, and η that infect either mucosal or cutaneous epithelia (Van Doorslaer et al., 2017; Zheng et al., 2022). Based on their oncogenic potential, mucosal HPVs can be categorized into low-risk (LR-) HPV types, which cause benign lesions, or high-risk (HR-) HPV types, which are involved in the development of cancer in the anogenital tract and oropharynx. Mucosal HR-HPV types such as 16 and 18 all belong to the genus α. There is also increasing evidence of an oncogenic role for certain cutaneotropic genus β-HPVs, i.e., a contribution to the development of cutaneous squamous cell carcinoma (cSCC) (Pfister, 2003; Hasche et al., 2018; Rollison et al., 2019; Lambert et al., 2020).

In patients with the rare genetic disorder epidermodysplasia verruciformis (EV), cSCCs develop at high rates (30%–60%) at sun-exposed sites (Jablonska and Majewski, 1994; Orth, 2006). Clinical data from EV patients and animal models suggest a co-carcinogenic role of genus β-HPVs, i.e., primarily HPV5 and 8, with ultraviolet (UV) radiation in skin carcinogenesis (summarized in (Venuti et al., 2019; Smola, 2020)). Already in EV precursor lesions, p53 mutations are detected (Padlewska et al., 2001), which is in strong contrast to cervical carcinogenesis following genus α HR-HPV infection, where p53 mutations are rarely found.

EV lesions express high levels of viral oncoproteins (Haller et al., 1995; Dell'Oste et al., 2009) actively modulating the local immune microenvironment. The HPV-infected epithelium typically lacks Langerhans cells, allowing the β-HPVs to establish persistence in EV patients. As the underlying mechanism, our previous studies had shown that the HPV8 E7 protein suppresses the Langerhans-attracting chemokine CCL20 by specifically targeting CCAAT/enhancer-binding protein β (C/EBPβ), a potent cellular inducer of differentiation-associated CCL20 transcription in human keratinocytes (Sperling et al., 2012).

Transgenic mouse models, further characterized the E6 protein as the major oncogene of HPV8 (Schaper et al., 2005; Marcuzzi et al., 2009), and there is also increasing evidence that HPV8 E6 modulates initial steps critical for skin carcinogenesis in EV patients. One early hallmark identified in EV lesions is the expansion of the p63-positive epithelial compartment. Based on sequence homologies, p63 belongs to the p53 family of transcription factors and is a major regulator of proliferation in the skin (Melino et al., 2015). Transcription from an alternative promoter results in the truncated ΔN isoform lacking the transactivation domain of p63, which can act in a dominant-negative manner (Yang et al., 1998; Westfall et al., 2003). ΔNp63 characterizes progenitor or stem cells in skin that are particularly prone to carcinogenesis (Missero and Antonini, 2017). Mechanistically, our previous studies have demonstrated that HPV8 E6 downregulates C/EBPα (Marthaler et al., 2017), a potent suppressor of UV-induced skin carcinogenesis (Schuster and Porse, 2006; Thompson et al., 2011) to prevent epithelial microRNA-203 induction. MiR-203 targets p63 and reduces its expression during skin differentiation (Yi et al., 2008). Conversely, HPV8 E6-mediated suppression of miR-203 leads to an increase of ΔNp63 (Marthaler et al., 2017). Moreover, HPV8 E6 interferes with UV-induced apoptosis (Underbrink et al., 2008) and with mastermind-like protein 1 (MAML1) to suppress Notch (Tan et al., 2012; Meyers et al., 2013). There is also increasing evidence that HPV8 E6 impairs DNA repair pathways (Hu et al., 2023) and facilitates the accumulation of UV-induced DNA mutations (summarized in (Wendel and Wallace, 2017; Rollison et al., 2019)).

A second hallmark found in EV patients is the infiltration of lesional skin with CD45-positive leukocytes, and the HPV8 E2 protein was shown to contribute to the upregulation of calgranulins S100A8 and S100A9 and neutrophil recruitment (Podgorska et al., 2018). Also in mice transgenic either for the HPV8 early region or the HPV8 E6 only, tumor formation was enhanced and accelerated not only by UV irradiation but also by skin wounding. In both scenarios, this was accompanied by strong inflammatory infiltrates (Schaper et al., 2005; Marcuzzi et al., 2009). Importantly, crossing K14-HPV8 E6 transgenic mice with mice lacking the chemokine receptor CCR2, which responds to the monocyte chemoattracting protein (MCP-1, CCL2), the key regulator of monocyte chemotaxis, prevented signs of epidermal hyperplasia and dysplasia after UV irradiation, suggesting a key role of monocyte recruitment in HPV8-associated skin carcinogenesis (Lelios et al., 2021).

Stromal monocyte recruitment has previously also been identified as a critical event in cervical carcinogenesis *in vivo* (Mazibrada et al., 2007), and these immune cells were shown to produce the angiogenesis and invasion promoting matrix-metalloproteinase MMP-9 (Schroer et al., 2011). Oncoproteins of mucosal genus α-HR-HPV suppress rather than induce CCL2 in infected keratinocytes (Kleine-Lowinski et al., 2003), and expression of this chemokine is still low in cervical cancer cells (Altenburg et al., 1999; Hess et al., 2000). Therefore, the mechanism underlying monocyte recruitment in cervical carcinogenesis remained unclear until HR-HPV-transformed cells were shown to induce CCL2 to enormous amounts in the stromal myeloid cell compartment in a paracrine, STAT3-dependent manner (Schroer et al., 2011). Molecular *in vitro* studies suggested that genus β-HPVs, however, might differ from genus α-HPVs in this regard. Rather a direct effect on CCL2 induction by HPV5 E6/E7 was observed, when both oncogenes were expressed together in normal human keratinocytes, and CCL2 protein expression was shown to be higher in the p53-mutated HaCaT keratinocyte cell line (Lehman et al., 1993; De Andrea et al., 2007).

To further analyze this intriguing aspect of genus β-HPV oncoproteins, we investigated EV lesions *in situ* and molecular mechanism *in vitro* using a broad multiplex approach in our HPV8 models. In this study, we show that genus β-HPV8 E6 but not the E7 oncoprotein significantly and strongly induces CCL2 in keratinocytes, leading to CCR2-dependent monocyte recruitment *in vitro*. Mechanistically, we provide evidence for a prominent role of the recently identified C/EBPα/miR-203/p63 pathway for HPV8 E6-mediated CCL2 induction. This pathway is functional in p53-mutated HaCaT keratinocytes, which were used a model for UV-damaged skin keratinocytes. The presence of macrophages and epithelial co-expression of p63 and CCL2 were confirmed in organotypic air–liquid-interface cultures *in vitro* and in lesional epidermis of EV patients *in situ*.

The results of this study demonstrate that both pro-carcinogenic functions of HPV8 E6, promotion of keratinocyte stemness and support of an inflammatory microenvironment, are mechanistically linked and depend on the downregulation of the tumor-suppressor C/EBPα and subsequent induction of the stemness-regulator ΔNp63.

## 2 Materials and methods

### 2.1 Ethical statement

The study was conducted according to the principles expressed in the Declaration of Helsinki. The use of human foreskin keratinocytes and human blood samples was approved by the local Ethics Committee of the Saarland (Ärztekammer des Saarlandes, Saarbrücken, Germany, votes 15/13 and 207/10). EV samples were identical to those used in Sperling et al. (2012) and Marthaler et al. (2017) provided by Magdalena Malejczyk and Prof. Sławomir Majewski, both Medical University of Warsaw, Warsaw, Poland. The use of EV skin had been approved by the Bioethics Committee at the Medical University of Warsaw, Poland. All human samples were analyzed anonymously.

### 2.2 Cell culture and transfection

NHK were isolated from foreskin tissue and cultivated in supplemented KGM2 medium (PromoCell, all material identifiers in **Supplementary Table 1**) and 100 µM CaCl_2_. HaCaT cells (a gift from Prof. P. Boukamp, German Cancer Research Center, Heidelberg, Germany (Boukamp et al., 1988) with mutated p53 alleles (Lehman et al., 1993)), and foreskin fibroblasts, isolated from foreskin tissue were cultivated in DMEM medium (PAN-Biotech) supplemented with 10% FCS and 1 mM sodium pyruvate. 100 µg/ml penicillin/streptomycin was used as antibiotic. HPV8 E6-, E7-, or E6/E7-expressing cells were generated by retroviral infection using the pLXSN vector system (Clontech) as described (Sperling et al., 2012; Marthaler et al., 2017). pLXSN plasmid-containing cells were selected with 100 µg/ml G-418. For TNF-α stimulation, 1.5 × 10^5^ cells were seeded into 12-well plates. Next day, cells were treated with 1,000 U TNF-α (Boehringer Ingelheim) or PBS as control and harvested after 16 h. Organotypic 3D cultures were generated by seeding 5 × 10^5^ foreskin fibroblasts (passages 3–7) in 4-mg/ml rat tail collagen into 12-well plates and culturing in DMEM medium. Next day, 7 × 10^5^ HaCaT pLXSN or HPV8 E6-expressing cells were added on top of the fibroblast containing collagen in a 1:4 mixture of Ham’s F12 medium and DMEM medium supplemented as described in Hadaschik et al. (2003). On day 3, the cultures were transferred on a metal grid in 6-well plates and were cultivated at the liquid–air interface for 7 days. 3D cultures were fixed in 4% paraformaldehyde and embedded in paraffin.

For knockdown experiments, 3 × 10^5^ HaCaT cells or 2 × 10^5^ NHK, respectively, were seeded into 6-well plates. Next day, cells were transfected with 10 nM siRNA directed against p63 and C/EBPα or control siRNA (sequences **Supplementary Table 2**) using 5 µl of Lipofectamine RNAiMAX reagent (Fisher Scientific).

### 2.3 Quantitative real-time PCR

RNA was extracted using the NucleoSpin RNA Kit (Macherey-Nagel), and reverse transcription was done with 500 ng RNA using Maxima Reverse Transcriptase (Thermo Fisher). qRT-PCR was performed using intron-spanning primers designed with the UPL software (Roche) (**Supplementary Table 3**), the Universal Probe System (Roche) and the LightCycler 480II (Roche). mRNA expression levels were determined in relation to RPL13A and calculated using the 2-ΔΔCt method.

### 2.4 Immunological detection methods

Protein extraction was performed with radioimmunoprecipitation (RIPA) buffer (Sigma). 15 µg protein was analyzed with 10% SDS gels and Western blot using rabbit anti-p63 (1:1,000, D9L7L, Cell Signaling), rabbit anti-C/EBPα (1:1,000, 56F10, Cell Signaling), mouse anti-β-actin (1:10,000, AC-15, Sigma), goat anti-rabbit-peroxidase (1:2,000, Sigma), rabbit anti-mouse-peroxidase (1:2,000, Sigma), and WesternBright ECL HRP substrate (Advansta). Chemiluminescence was detected with Azure 500 (Biozym). Protein expression levels were normalized to β-actin expression levels. Immunohistochemical (IHC) staining of FFPE sections was done as previously described (reagents in **Supplementary Table 1**) (Marthaler et al., 2017). HPV-8-positive EV specimens were identical to those previously published (Sperling et al., 2012; Marthaler et al., 2017). CCL2 protein concentrations in cell supernatants were measured with ELISA MAX Deluxe Set Human MCP-1/CCL2 (BioLegend) according to the manufacturer’s specifications and the Mini ELISA Plate Reader (BioLegend). Multiplex immunoassays were performed using the Bio-Plex Pro Human Cytokine Screening Panel (48-Plex, **Supplementary Table 4**) (Bio-Rad) according to the manufacturer’s specifications and the Bio-Plex 200 system (Bio-Rad). For conditioned media, 1.5 × 10^5^ pLXSN or HPV8 E6-expressing HaCaT cells were seeded into 12-well plates and harvested after 16 h.

### 2.5 Migration assay

CD14^+^ monocytes were isolated from PBMCs of peripheral blood of healthy donors by density gradient centrifugation using Pancoll 1.077 g/ml (PAN-Biotech) and a subsequent magnetic cell sorting (MACS) using anti-CD14-coupled magnetic beads (Miltenyi Biotec) and the MidiMACS Separator (Miltenyi Biotec). Purity of freshly isolated CD14^+^ cells was determined by direct immunofluorescence using a fluorescein (FITC)-labeled mouse anti-human-CD14 antibody (1:10, M5E2, BD Biosciences), monocyte viability with 500 ng/ml 7-AAD, and flow cytometry (**Supplementary Figure S1**) using a FACSCanto II (BD Biosciences) and FACSDiva software (version 8.0.1). Migration assay was performed by adding 5 × 10^5^ CD14^+^ cells on a Transwell with 5-µm pore size in a 24-well plate (Corning) in RPMI medium supplemented with 10% FCS and 1 mM sodium pyruvate. CD14^+^ cells were exposed to conditioned media derived from pLXSN- or HPV8 E6-expressing HaCaT cells. For conditioned media, 6 × 10^5^ cells were seeded into 6-cm plates and harvested after 16 h. In indicated experiments, CD14^+^ cells were pretreated for 1 h with 10 µM, 30 µM, and 50 µM RS102895 or DMSO as control and their viability was measured using 7-AAD (BioLegend). After 4 h, migrated cells were fixed with 70% ethanol and stained with 0.2% crystal violet solution. Migrated cells of six independent areas were counted under a microscope with 20× magnification and documented.

### 2.6 Statistics

Data were generated from at least three independent experiments if not otherwise indicated. Graphical, correlation, and statistical analyses were performed using GraphPad Prism 9.3.1. (GraphPad Software, San Diego, USA). Group data are reported as mean ± SEM. Data of multiple experiments were illustrated as bar blots showing individual results. Significance was determined by two-tailed unpaired t-test to compare the mean of two groups, with one-way ANOVA and Tukey correction or two-way ANOVA and Sidak correction for multiple comparison. Significance was accepted when p-values were ≤0.05.

## 3 Results

### 3.1 HPV8 E6 but not E7 significantly induces CCL2 expression in HaCaT keratinocytes

**Figure 1:**
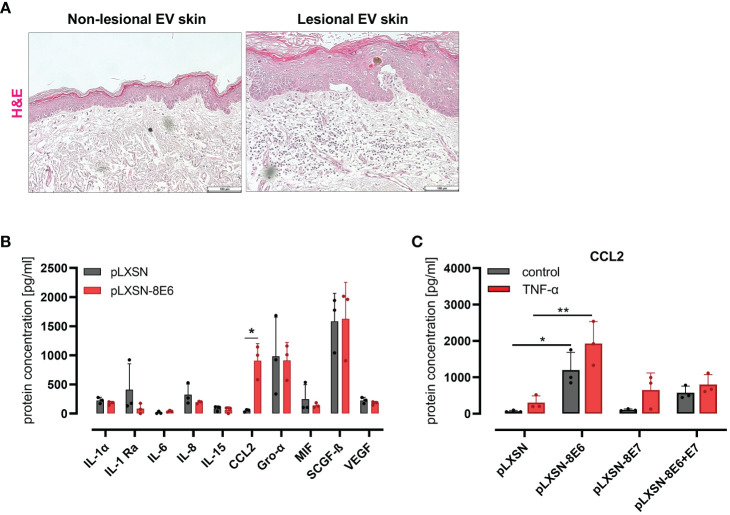
HPV8 E6 but not E7 induces CCL2 expression in HaCaT keratinocytes **(A)** H&E staining of non-lesional and lesional EV skin. Scale bars 100 µm. Representative image of n = 3. **(B)** Cytokine detection by 48-plex array in conditioned media of HaCaT cells after retroviral gene transfer of HPV8 E6 or empty plasmid (pLXSN) (full list: **Supplementary Table 4**). **(C)** CCL2 protein expression by ELISA of HaCaT cells after retroviral gene transfer of HPV8 E6 or/and E7 or empty plasmid (pLXSN) and 16-h TNF-α stimulation (1,000 U/ml). Data are representative images of n = 3 in A or results of three individual experiments in **(B, C)** and presented as “protein concentration [pg/ml]” in **(B)** Significant differences were calculated with two-way ANOVA and Sidak correction for multiple comparison and displayed as *p < 0.05, **p < 0.01.

Lesional skin of EV patients, but not the non-lesional skin, is highly infiltrated with immune cells (**Figure 1A**), previously shown to be CD45-positive leukocytes (Podgorska et al., 2018).

To identify inflammatory cytokines and chemokines regulated by the HPV8 E6 protein in an unbiased approach, we used HaCaT cells as a model for UV-damaged skin keratinocytes. This spontaneously immortalized skin keratinocyte cell line displays p53 mutations typical of UV light-induced mutations (Lehman et al., 1993) as expected in sun-exposed EV lesions. Media conditioned from HaCaT keratinocytes engineered by retroviral gene transfer to express HPV8 E6 (S1 C) or pLXSN empty vector control cells were compared for cytokine protein expression using a multiplex immunoassay (48-panel, **Supplementary Table 4**). There were 10 cytokines detected above a threshold of 30 pg/ml (**Figure 1B**). Among these, only the expression of CCL2 was strongly and significantly (p = 0.0135) produced by HPV8 E6-expressing HaCaT cells (914 ± 292 pg/ml) as compared with control cells (52 ± 22 pg/ml). Other cytokines that were expressed at lower (IL-1α, IL-1ra, IL-6, IL-8, IL-15, MIF, VEGF) or higher levels (Gro-α, SCGF-β) were not significantly induced by HPV8 E6.

CCL2 induction had previously been demonstrated in keratinocytes expressing HPV5 E6/E7 (De Andrea et al., 2007). To address a potential role for HPV8 E7 in the absence or presence of E6, we compared CCL2 in HaCaT cells transduced to express HPV8 E6, E7, or E6/E7 together (S1 C) by qRT-PCR and ELISA. Compared with pLXSN control cells, CCL2 protein was significantly induced in E6, weakly in E6/E7 expressing keratinocytes but not in cells expressing E7 alone (**Figure 1C**). Treatment with 1,000 U/ml TNF-α, a potent proinflammatory cytokine and well-described inducer of CCL2 (Zachariae et al., 1990), increased CCL2 in all conditions tested. Notably, in HPV8 E6-expressing HaCaT cells, CCL2 expression was significantly higher than in pLXSN control cells treated with TNF-α.

### 3.2 HPV8 E6 expression induces CCR2-dependent monocyte migration *in vitro* corresponding to macrophage infiltrates in EV lesions *in vivo*

Recent data suggested a substantial role of CCR2-dependent monocyte recruitment in HPV8-associated skin carcinogenesis (Lelios et al., 2021). Therefore, we were interested in whether HPV8 E6-expressing HaCaT cells were sufficient to stimulate monocyte migration and whether this was dependent on the CCL2/CCR2 axis.

**Figure 2:**
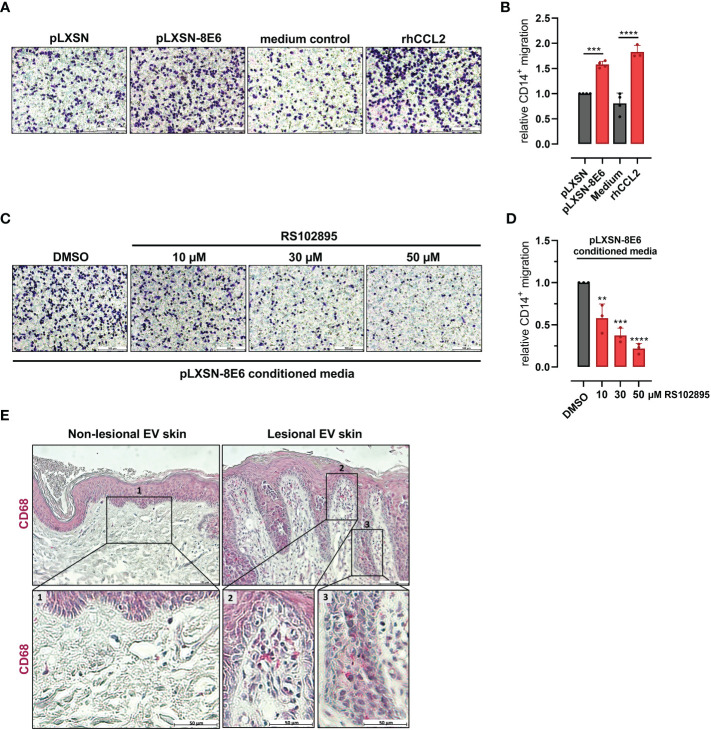
HPV8 E6-dependent monocyte migration and presence of CD68-positive immune cells in EV skin: **(A)** Crystal violet stained monocytes migrated after stimulation with conditioned media from HaCaT cells transduced with HPV8 E6 or empty pLXSN vector, medium control, or 1 µg/ml rhCCL2. Scale bars 100 µm. Representative images of n = 3. **(B)** Cells of three independent experiments in A were counted. **(C)** Monocytes were stimulated with conditioned media from HPV8 E6-transduced HaCaT cells and treated with CCR2-specific antagonist RS102895 at the indicated concentrations or vehicle control. Scale bars 100 µm. Representative images of n = 3. **(D)** Cells of three independent experiments in C were counted. Calculations are presented as “relative CD14^+^ migration” in B and **(D)** Significant differences were calculated with one-way ANOVA and Tukey correction for multiple comparison and displayed as **p < 0.01, ***p < 0.001, and ****p < 0.0001. **(E)** IHC of FFPE sections from non-lesional and lesional EV skin using human CD68-specific antibody and hematoxylin counterstaining. Scale bars 100 µm. Representative image of n = 3. Magnification of non-lesional tissue (see insert 1), stromal or epidermal sections of lesional EV skin (see insert 2 or 3) is shown in the lower panel, scale bars 50 µm.

Cells positive for CD14, a cell surface marker expressed on monocytes and macrophages, isolated from peripheral blood of healthy donors were treated with conditioned media from HPV8 E6-expressing HaCaT or pLXSN control cells. Monocyte migration through Transwells was visualized and quantified after crystal violet staining. Media conditioned by HPV8 E6 expressing cells induced monocyte migration significantly more (1.6 ± 0.07-fold, p = 0.0002) than media from control cells (**Figures 2A, B**). HPV8 E6-dependent monocyte migration was similarly strong as with 1 µg/ml recombinant human (rh) CCL2 (1.8 ± 0.13-fold) used as a positive control.

Pretreatment of CD14-positive cells with the CCR2-specific antagonist RS102895 (10 µM, 30 µM, and 50 µM; (Schroer et al., 2011)) significantly (p = 0.0036, p = 0.0002, and p < 0.0001 for 10 µM, 30 µM, and 50 µM, respectively) impaired monocyte migration induced by conditioned media from HPV8 E6-expressing HaCaT cells in a dose-dependent manner, highlighting the role of the CCL2/CCR2 axis (**Figures 2C, D**).

To address the presence of monocyte-derived cells *in vivo*, we stained HPV8-positive skin sections of EV patients for CD68, a marker for macrophages. Lesional skin showed a prominent stromal and epithelial infiltration with CD68-positive cells, which was not observed in non-lesional skin (**Figure 2E**).

### 3.3 C/EBPα and p63 modulate CCL2 expression in an opposing manner

**Figure 3:**
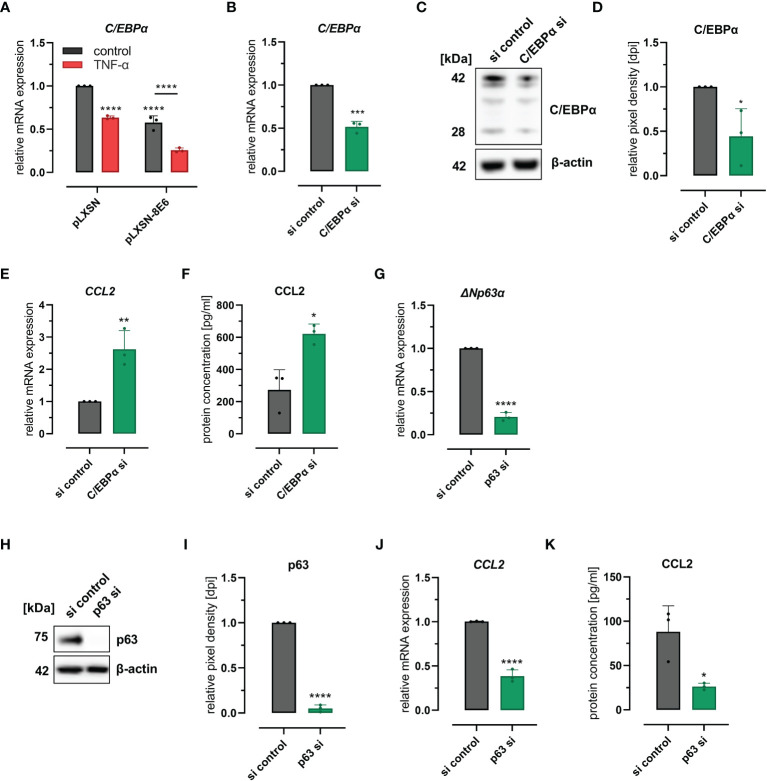
C/EBPα- and p63-dependent regulation of CCL2: **(A)** Expression of CEBPα mRNA in HaCaT cells transduced with HPV8 E6 or empty pLXSN vector and treated with TNF-α or medium control. **(B–F)** HaCaT cells were treated with a pool of four siRNAs targeting CEBPα or control siRNAs (si). Expression of CEBPα was detected by qRT-PCR **(B)**, immunoblot (C, representative image), densitometry of three independent experiments **(D)**, and of CCL2 using qRT-PCR **(E)** and ELISA **(F)** of respective conditioned media. **(G–K)** HaCaT cells were treated with a pool of four siRNAs targeting p63 or control siRNAs. Expression of ΔNp63α-specific mRNA was detected by qRT-PCR **(G)**, of total p63 protein by immunoblot (H, representative images, I, densitometry of three independent experiments), and of CCL2 mRNA by qRT-PCR **(J)** and protein **(K)** using ELISA and respective conditioned media. Results are demonstrated as “relative mRNA expression” in **(A, B, E, G, J)** normalized to RPL13A housekeeping gene expression, as “relative pixel density [dpi]” calculated by ImageJ in D and I, and as “relative protein concentration [pg/ml]” in F and **(K)** Significant differences were calculated with two-way ANOVA and Sidak correction for multiple comparison **(A)** and with two-tailed unpaired t-test **(B–K)** and displayed as *p < 0.05, **p < 0.01, ***p < 0.001 and ****p < 0.0001.

Recently, we have shown that HPV8 E6 suppresses the transcription factor C/EBPα, which we identified as a novel regulator of miR-203 that controls suprabasal p63 expression in skin (Marthaler et al., 2017). It is also known that C/EBPα is repressed by TNF-α (Stephens and Pekala, 1991; Yin et al., 1996), a potent inducer of CCL2 expression in various cell types (Zachariae et al., 1990). We were therefore interested to explore a role of C/EBPα in HPV8 E6-mediated CCL2 induction. First, we confirmed the repressive effect of HPV8 E6 and TNF-α on C/EBPα expression in HaCaT cells. C/EBPα mRNA expression was significantly reduced in cells treated with TNF-α (37%, 0.63 ± 0.02) or in cells expressing HPV8 E6 (42%, 0.58 ± 0.08) (**Figure 3A**). The effect on C/EBPα was even more pronounced, when HPV8 E6-expressing cells were treated with TNF-α (74%, 0.36 ± 0.03) (HPV8 E6 vs. pLXSN untreated: p < 0.0001, pLXSN untreated vs. treated: p < 0.0001, HPV8 E6 untreated vs. TNF-α treated p < 0.0001).

To evaluate a mechanistic role of C/EBPα and p63 for CCL2 expression, we performed knockdown experiments in HaCaT wild-type cells. Transfection of C/EBPα siRNAs resulted in 48% (0.52 ± 0.06) and 56% (0.44 ± 0.31) efficiency at mRNA and protein levels as quantified by qRT-PCR and Western blot (**Figures 3B–D**), respectively. C/EBPα siRNAs significantly (p = 0.0083 and p = 0.0122) increased CCL2 mRNA (2.6 ± 0.58-fold) and protein (2.7 ± 1.14-fold) expression, levels in HaCaT cells as measured by qRT-PCR and ELISA (**Figures 3E, F**). Transfection of p63 siRNAs reduced p63 expression by 79% (0.21 ± 0.05) and 95% (0.05 ± 0.04) at mRNA (ΔNp63) and protein levels, respectively (**Figures 3G–I**) and significantly (p = 0.0001 and p = 0.0226) reduced CCL2 mRNA (61%, 0.39 ± 0.07) and protein expression (58%, 0.42 ± 0.07) (**Figures 3J, K**). Single siRNAs specific for p63 mRNA further supported these findings (S2 A-E). These data also demonstrated that CCL2 regulation was dependent on the efficacy of the respective p63 siRNA. Together, these results indicated that the C/EBPα-p63 pathway can modulate CCL2 chemokine expression.

### 3.4 HPV8 E6 induces CCL2 expression via the C/EBPα/mir-203/p63 pathway

**Figure 4:**
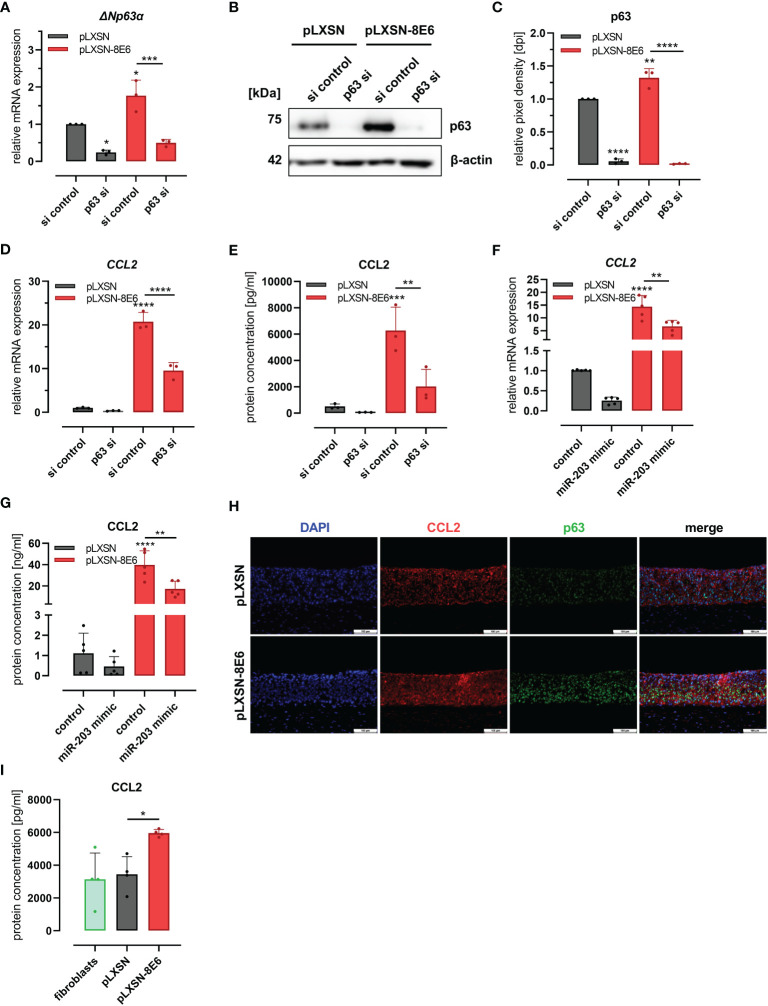
HPV8 E6 involves the C/EBPα/p63/mir-203 pathway to induce CCL2: **(A–E)** HaCaT cells transduced to express HPV8 E6 (red) or empty vector (gray) were treated with a pool of four siRNAs targeting p63 or control siRNAs (si). Expression of p63 was investigated by qRT-PCR (**A**, ΔNp63α mRNA) and immunoblot **(B)** representative images, **(C)**, densitometry of three independent experiments) and of CCL2 mRNA using qRT-PCR **(D)** and protein by ELISA **(E)** in respectively conditioned media. HaCaT cells transduced with HPV8 E6 (red) or empty pLXSN vector (gray) were treated with miR-203 mimic or control. CCL2 mRNA was detected by qRT-PCR **(F)** and protein using ELISA **(G)**. **(H)** Indirect immunofluorescence of CCL2 (red) and p63 (green) in 3D cultures of HaCaT cells transduced with HPV8 E6 or empty pLXSN vector. Scale bars 100 µm. Representative image of n = 3. **(I)** CCL2 content of media conditioned by organotypic 3D cultures of fibroblasts (green) and with HaCaT cells transduced with 8E6 (red) or empty vector (gray) was detected by ELISA in three independent experiments. Results are demonstrated as “relative mRNA expression” in **(A, D, F)** normalized to RPL13a housekeeping gene expression, as “relative pixel density [dpi]” calculated by ImageJ in C, and as “relative protein concentration [pg/ml]” in E and I, [ng/ml] in G. Significant differences were calculated with two-way ANOVA and Sidak correction **(A–G)** and one-way ANOVA and Tukey correction **(I)** for multiple comparison and displayed as *p < 0.05, **p < 0.01, ***p < 0.001, and ****p < 0.0001.

To further investigate whether this C/EBPα- and p63-dependent mechanism was involved in HPV8 E6-mediated induction of CCL2, we performed the siRNA experiments also in HPV8 E6-expressing HaCaT cells and pLXSN vector controls (**Figures 4A–E**). The expression of p63 was significantly (p = 0.0106 and p = 0.0027) increased in HPV8 E6-expressing cells transfected with control siRNAs, at both mRNA (1.77 ± 0.42-fold) and protein levels (1.32 ± 0.01-fold) (**Figures 4A–C**). Transfection of p63 siRNAs led to a significant reduction of p63 expression by 50%–75% on mRNA (ΔNp63α) and up to 98.5% on protein level in pLXSN- and HPV8 E6-expressing cells (mRNA: pLXSN p = 0.0114, HPV8 E6 p = 0.0004; protein: pLXSN and HPV8 E6 p < 0.0001). The suppression of p63 resulted in a reduced expression of CCL2 in pLXSN HaCaT cells on mRNA and protein levels as measured by qRT-PCR and ELISA (**Figures 4D, E**). Induction of CCL2 mRNA (20.74 ± 2.13-fold) and protein expression (6217 ± 1179 pg/ml) in HPV8 E6-expressing cells was significantly reduced by 54% on mRNA level (p < 0.0001) and down to 2,026 ± 1,306 pg/ml at the protein level (p < 0.0093) upon p63 siRNA transfection. Treating HaCaT cells with single siRNAs specific for p63 mRNA yielded similar results (S2 D and E).

To further explore the role of miR-203 in CCL2 regulation, we transfected a miR-203 mimic into pLXSN and HPV8 E6-expressing HaCaT cells. MiR-203 overexpression in pLXSN-expressing cells led to a decreased CCL2 expression at the mRNA (0.26 ± 0.1) and protein levels (0.46 ± 0.49, **Figures 4F, G**), similar to p63 siRNA transfection. The strong effect of HPV8 E6 on CCL2 mRNA (13 ± 5.28) and protein induction (39.8 ng/ml ± 13.07) was significantly counteracted by miR-203 expression by 56.6% (5.64 ± 2.27 and 17.17 ng/ml ± 7.35 respectively, p = 0.0064 and 0.0013).

We then used HPV8 E6-expressing and pLXSN-transduced control HaCaT cells to generate organotypic 3D cultures (**Figure 4H**). As shown by immunofluorescence co-staining, the induction of CCL2 expression in HPV8 E6-expressing 3D epithelia was associated with a pronounced expression p63. Conditioned media of the HPV8 E6-expressing 3D cultures contained a significantly (p = 0.0277) higher amount of CCL2 protein (5,963 ± 217 pg/ml) compared with those with pLXSN vector control (3,449 ± 1,076 pg/ml) or fibroblast 3D cultures without keratinocytes (3,140 ± 1,601 pg/ml) (**Figure 4I**).

Together, these data suggest that the C/EBPα/miR-203/p63 pathway is involved in HPV8 E6-mediated induction of the CCL2 chemokine.

### 3.5 HPV8 E6-mediated CCL2 expression in normal keratinocytes involves p63, which is co-expressed with CCL2 in lesional EV skin

**Figure 5:**
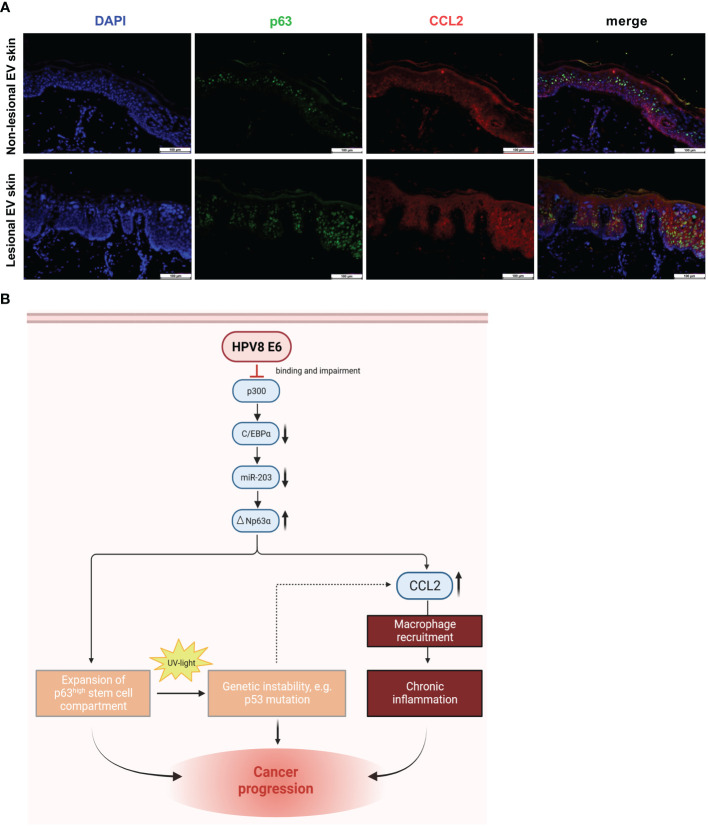
Co-expression of p63 and CCL2 in lesional *EV* epidermis and graphical summary of the underlying pathway identified *in vitro* with potential consequences *in vivo*: **(A)** Indirect immunofluorescence on non-lesional and lesional EV-skin using human p63-specific (green) and CCL2-specific (red) antibody on FFPE slides. Scale bars 100 µm. Representative image of n = 3. **(B)** Schematic representation of the HPV8 E6-induced C/EBPα/miR-203/p63 pathway, mechanistically linking epithelial stemness and inflammation, both of which potentially contribute to skin cancer progression in EV patients. Expansion of the ΔNp63-positive stem cell compartment increases UV susceptibility, and CCL2 induction promotes an inflammatory microenvironment, a hallmark of cancer (Hanahan and Weinberg, 2011). Created with BioRender.com.

To validate whether CCL2 expression is associated with p63 expression *in vivo*, we analyzed CCL2 and p63 protein expression in HPV8-positive EV specimens by co-immunofluorescence staining (**Figure 5A**). In lesional EV epithelia, we indeed observed a suprabasal overlapping expression pattern of p63 and CCL2. In contrast, in non-lesional skin, suprabasal CCL2 expression was much weaker. This corresponded to the expansion of the p63-expressing epithelial compartment in β-HPV-positive skin lesions as shown previously (Marthaler et al., 2017).

In summary, our data support a role of the stemness-regulating p63-pathway for HPV8-induced CCL2 upregulation, which may contribute to the inflammatory microenvironment observed in EV lesions.

## 4 Discussion

In the present study, we have shown for the first time that the monocyte attracting chemokine CCL2 is strongly upregulated in the lesional epidermis of EV patients, and we have confirmed the presence of macrophages in the corresponding stromal and, to a lesser extent, also in the epidermal compartment. Mechanistically, we have demonstrated that HPV8 E6-expressing HaCaT keratinocytes are capable to attract monocytes via the CCL2/CCR2 axis and that the recently identified C/EBPα/miR-203/p63 pathway plays an important role for CCL2 upregulation in the HPV8 E6-expressing keratinocytes. Our data provide evidence for a novel molecular mechanism driven by HPV8 E6 to actively modulate the local immune microenvironment, potentially paving the way for carcinogenesis in EV patients (**Figure 5B**).

Stromal infiltration with inflammatory monocytes, which we have detected in EV lesions, has previously also been observed in cervical carcinogenesis. Infiltrates culminated at the stage of high-grade lesions (al-Saleh et al., 1995; Schroer et al., 2011). However, in mucosal genus α HR-HPV-associated carcinogenesis CCL2 was induced via a mechanism differing from cutaneous genus β-HPVs. Oncoproteins from mucosal high-risk HPV types rather suppress CCL2 expression (Kleine-Lowinski et al., 2003; De Andrea et al., 2007) and other proinflammatory responses in normal human keratinocytes (Karim et al., 2011, 2013). Detailed analyses revealed that during cervical carcinogenesis, CCL2 is massively induced in myelomonocytic cells in a paracrine manner by HPV-transformed cells. Mechanistically, this involved a STAT3-dependent signaling pathway and led to the expression of the pro-tumorigenic matrix-metalloproteinase MMP-9 in the myeloid cells (Schroer et al., 2011).

In contrast, HPV5 E6/E7 (De Andrea et al., 2007) or HPV8 E6 alone (as shown here) promoted CCL2 upregulation in the host keratinocytes. HPV8 E6 led to a strong transcriptional induction of CCL2 in HaCaT keratinocytes, and this was also observed in normal human keratinocytes retrovirally transduced to express HPV8 E6 (S3 A and B). Interestingly, CCL2 protein was detectable in higher quantities only in the supernatants of the HPV8 E6-expressing HaCaT cells but not in those of the normal human keratinocytes (data not shown) corresponding to the observations made for HPV5 E6/E7 (De Andrea et al., 2007). HaCaT cells are skin-derived keratinocytes with mutated *TP53* alleles (Lehman et al., 1993). *TP53* genes are classical targets of UV photocarcinogenesis (Ziegler et al., 1994), and mutations occur as a result of UV-induced pyrimidine–pyrimidone photoproducts and unrepaired cyclobutane pyrimidine dimers. UV signature mutations do not only play a role for skin carcinogenesis of the normal population, but they are also found in precursor lesions of EV patients (Padlewska et al., 2001). Therefore, it can be speculated that the genus β-HPV E6 protein, which upregulates CCL2 at the transcriptional level, is further supported by the intracellular conditions of p53-mutated keratinocytes to fully promote CCL2 protein production (**Figure 5B**). This hypothesis would fit with the assumption that CCL2 production, and thus the inflammatory microenvironment, is enhanced by the β-HPV E6 protein particularly in skin areas that had previously been damaged by UV radiation. Moreover, it will be interesting to study in the future whether or not acute UV radiation of primary keratinocytes expressing HPV8 E6 would also lead to increased CCL2 production.

Notably, HPV8 E6 is not the only activator of innate inflammatory responses. We have recently demonstrated that genus β-HPV E2, another early protein, supports neutrophil recruitment via the calgranulins S100A8 and S100A9 (Podgorska et al., 2018). Why β-HPVs directly promote innate inflammatory responses supposed to activate the adaptive immune system, and how the viruses cope with this, can only be speculated. β-HPVs are adapted to UV irradiation and exploit UV-induced inflammatory signaling via AP-1 or interferon regulatory factor 7, to promote their life cycle (Stubenrauch et al., 1992; Akgul et al., 2005; Oldak et al., 2011). On the other hand, through expression of the E7 protein, HPV8 represses the chemokine CCL20 responsible for Langerhans cell recruitment to the epithelium. As a consequence, productive EV lesions are almost devoid of Langerhans cells, which may prevent antigen presentation in the epithelium (Sperling et al., 2012), and potentially also subsequent steps activating adaptive immunity.

As a side effect, chronic inflammation, a hallmark of cancer (Hanahan and Weinberg, 2011), could promote the malignant progression of benign lesions in EV patients. In our study, HPV8 E6 induced the recruitment of CD14-positive monocytes *in vitro* via the CCL2/CCR2 axis, as demonstrated with the inhibitor RS102895, which specifically blocks the CCL2 receptor CCR2 (Schroer et al., 2011). Other chemotactic cytokines for monocyte recruitment (MCP-3, MIP1α, MIP1β, RANTES, M-CSF, and GM-CSF (Pollard, 2004) were not detectable by cytokine profiling. The *in vivo* importance of this CCL2/CCR2 axis was recently demonstrated in a K14-HPV8 E6 transgenic mouse model, in which cSCC occurs spontaneously at a rate of 6% and is strongly enhanced and accelerated by UV irradiation or skin wounding (Marcuzzi et al., 2009). HPV8 E6-expressing mice crossed to *Ccr2^−/−^* mice lacked lesional monocyte infiltration and were shown to be resistant to UV-induced cSCC formation (Lelios et al., 2021). This strongly supported the notion that recruited monocytes were required for HPV8 E6-driven tumor development.

Our *in vitro* experiments demonstrated that CCL2 induction by HPV8 E6 was even stronger than stimulation with the proinflammatory cytokine TNF-α. TNF-α can suppress C/EBPα in various tissues (Stephens and Pekala, 1991; Yin et al., 1996), a transcription factor which we recently identified as a target of HPV8 E6 signaling (Marthaler et al., 2017). C/EBPα is a tumor suppressor in multiple tissues (Schuster and Porse, 2006) and is involved in UV-induced skin carcinogenesis (Thompson et al., 2011). Our previous studies identified C/EBPα as a critical regulator of miR-203 (Marthaler et al., 2017), which controls suprabasal expression of the stemness regulator ΔNp63α, proliferation, and epidermal differentiation (Lena et al., 2008; Yi et al., 2008).

In the present study, we have demonstrated that HPV8 E6 involves this recently identified C/EBPα/miR-203/p63 pathway for CCL2 induction using siRNA and microRNA mimic. Despite similar potency of C/EBPα downregulation, HPV8 E6-expressing cells always produced higher amounts than C/EBPα knockdown cells. Our study therefore does not exclude the contribution of other molecular pathways to HPV8 E6-mediated CCL2 induction. In the lesional EV epithelium, we have previously demonstrated a strong suprabasal upregulation of p63 (Marthaler et al., 2017), and this corresponded to an induction of CCL2 as shown here. ΔNp63α, lacking the N-terminal transactivation domain, is the predominant isoform of p63 in keratinocytes. Our findings correspond very well to previous reports showing that the stemness regulator ΔNp63α plays a significant role in orchestrating the cross-talk between neoplastic cells and the microenvironment of SCCs (Gatti et al., 2019) qualifying it as an oncogenic driver of SCCs (Riege et al., 2020). ΔNp63α has previously been demonstrated to regulate cytokine expression (Yang et al., 2011). Beyond that, in HPV16-associated penile carcinoma, we have recently shown that elevated p63 levels are associated with increased production of the neutrophil-recruiting chemokine CXCL8 (Bernhard et al., 2021). p63 contributed to the E6-induced CCL2 mRNA induction also in primary human keratinocytes. Treatment with p63-specific siRNA reduced CCL2 mRNA induction mediated by HPV8 E6 to basal levels comparable with pLXSN control cells (S3 C-F). P63 is a member of the p53 family. Both transcription factors p63 and p53 share overlapping response elements (Riege et al., 2020), and mutations in *TP53* can have a critical impact on the overall complex interplay between p53 family members and their activities (Stindt et al., 2015). Our data clearly demonstrate that the C/EBPα/miR-203/p63 pathway plays an important role for HPV8 E6-mediated CCL2 transcription also in the keratinocyte cell line HaCaT, which harbors mutated *TP53* genes (Lehman et al., 1993). Moreover, since TNF-α alone also suppresses C/EBPα, it is reasonable to assume that the C/EBPα/miR-203/p63 pathway could also contribute to CCL2 induction and myeloid cell recruitment in the absence of viral proteins but presence of, e.g., TNF-α only.

In summary, our data provide evidence that two pro-carcinogenic functions of the genus β-HPV type 8, to promote keratinocyte stemness and to support an inflammatory microenvironment, are mechanistically linked. Both depend on the E6-mediated downregulation of the tumor-suppressor C/EBPα/miR-203 pathway and subsequent induction of the stemness-regulator p63. Particularly in EV patients with persistent genus β-HPV infection, this inflammatory pathway could further intensify once p53 is mutated and pave the way for carcinogenesis (**Figure 5B**).

## Data availability statement

The raw data supporting the conclusions of this article will be made available by the authors, without undue reservation.

## Ethics statement

The studies involving humans were approved by the local Ethics Committee of the Saarland and by the Bioethics Committee at the Medical University of Warsaw, Poland. All human samples were analyzed anonymously. The studies were conducted in accordance with the local legislation and institutional requirements. Written informed consent for participation in this study was provided by the participants’ legal guardians/next of kin.

## Author contributions

LV: Conceptualization, Formal analysis, Investigation, Methodology, Visualization, Writing – original draft. AS: Conceptualization, Formal analysis, Investigation, Methodology, Validation, Visualization, Writing – original draft. SL: Conceptualization, Methodology, Supervision, Visualization, Writing – original draft. AF: Investigation, Methodology, Writing – review & editing. TS: Investigation, Methodology, Writing – review & editing. CW: Investigation, Writing – review & editing. MS: Resources, Writing – review & editing. TV: Resources, Writing – review & editing. KR: Resources, Writing – review & editing. MO: Methodology, Supervision, Writing – review & editing. SS: Conceptualization, Funding acquisition, Project administration, Supervision, Validation, Writing – original draft.
