# Supplementary material for: The cutaneous beta human papillomavirus type 8 E6 protein induces CCL2 through the CEBPα/miR-203/p63 pathway to support an inflammatory microenvironment in epidermodysplasia verruciformis skin lesions
Source: Front Cell Infect Microbiol. 2024 Mar 6;14:1336492. doi: 10.3389/fcimb.2024.1336492 (PMC10953690; doi:10.3389/fcimb.2024.1336492)
Supplement: Supplementary file 1 [file Image_1.pdf]

The cutaneous beta human papillomavirus type 8 E6 protein induces CCL2 through the CEBPα/miR-203/p63 pathway to support an inflammatory microenvironment in epidermodysplasia verruciformis skin lesions

**Luca Vella[1], Anna Sternjakob[1], Stefan Lohse[1], Alina Fingerle[1], Tanya Sperling[2], Claudia Wickenhauser[3], Michael Stöckle[4], Thomas Vogt[5], Klaus Roemer[6], Monika Ołdak[1,7], Sigrun Smola[1,8]*

[1]Institute of Virology, Saarland University Medical Center, Homburg/Saar, Germany

[2]Institute of Virology, University of Cologne, Cologne, Germany

[3]Institute of Pathology, University of Cologne, Cologne, Germany

[4]Department of Urology and Pediatric Urology, Saarland University Medical Center, Homburg/Saar, Germany

[5]Department of Dermatology, Saarland University Medical Center, Homburg/Saar, Germany

[6]Jose Carreras Center for Immune and Gene therapy, Saarland University Medical Center, Homburg/Saar, Germany

[7]Department of Histology and Embryology, Medical University of Warsaw, Warsaw, Poland

[8]Helmholtz Institute for Pharmaceutical Research Saarland (HIPS), Helmholtz Centre for Infection Research, Saarbrücken, Germany

*** Correspondence:**
Sigrun Smola
sigrun.smola@uks.eu

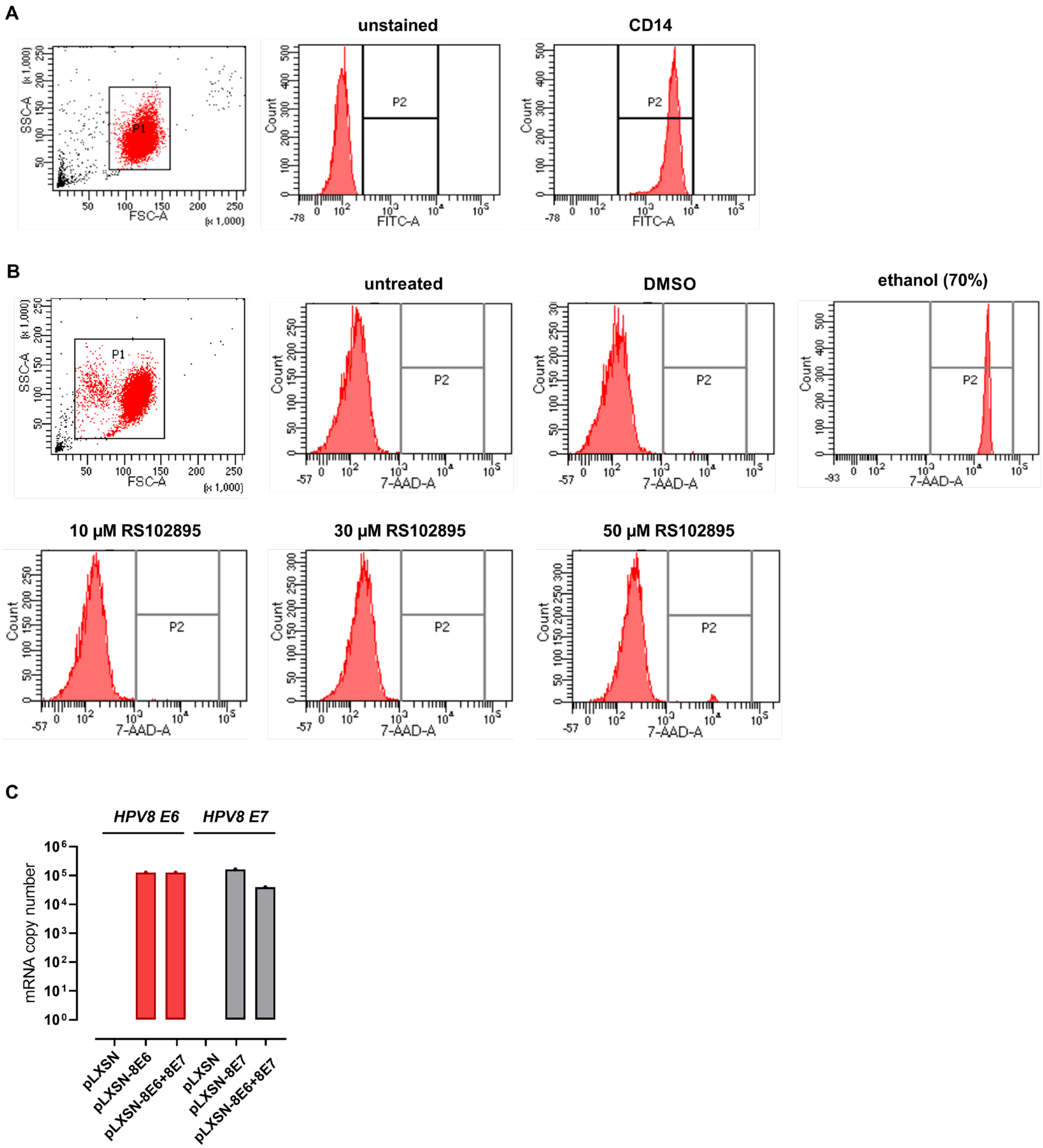

Supplementary Figure 1. A) Quantification of CD14 expressing monocytes by flow cytometry after purification from peripheral blood. B) Cell viability of $CD14^{+}$ monocytes after treatment with RS102895 (10 µM, 30 µM, and 50 µM), DMSO (negative control), or 70% ethanol (positive control) by 7-AAD uptake and subsequent FACS analysis. C) HPV8 E6 and E7 expression by qRT-PCR of HaCaT cells after retroviral gene transfer of pLXSN-HPV8 E6, E7, E6/E7 or empty plasmid (pLXSN).

Results demonstrated as “mRNA copy number” and were normalized to RPL13a housekeeping gene expression.
