# Supplementary material for: The cutaneous beta human papillomavirus type 8 E6 protein induces CCL2 through the CEBPα/miR-203/p63 pathway to support an inflammatory microenvironment in epidermodysplasia verruciformis skin lesions
Source: Front Cell Infect Microbiol. 2024 Mar 6;14:1336492. doi: 10.3389/fcimb.2024.1336492 (PMC10953690; doi:10.3389/fcimb.2024.1336492)
Supplement: Supplementary file 2 [file Image_2.pdf]

The cutaneous beta human papillomavirus type 8 E6 protein induces CCL2 through the CEBPα/miR-203/p63 pathway to support an inflammatory microenvironment in epidermodysplasia verruciformis skin lesions

Luca Vella[1], Anna Sternjakob[1], Stefan Lohse[1], Alina Fingerle[1], Tanya Sperling[2], Claudia Wickenhauser[3], Michael Stöckle[4], Thomas Vogt[5], Klaus Roemer[6], Monika Ołdak[1,7], Sigrun Smola[1,8]*

[1]Institute of Virology, Saarland University Medical Center, Homburg/Saar, Germany

[2]Institute of Virology, University of Cologne, Cologne, Germany

[3]Institute of Pathology, University of Cologne, Cologne, Germany

[4]Department of Urology and Pediatric Urology, Saarland University Medical Center, Homburg/Saar, Germany

[5]Department of Dermatology, Saarland University Medical Center, Homburg/Saar, Germany

[6]Jose Carreras Center for Immune and Gene therapy, Saarland University Medical Center, Homburg/Saar, Germany

[7]Department of Histology and Embryology, Medical University of Warsaw, Warsaw, Poland

[8]Helmholtz Institute for Pharmaceutical Research Saarland (HIPS), Helmholtz Centre for Infection Research, Saarbrücken, Germany

Supplementary Material

*** Correspondence:**
Sigrun Smola
sigrun.smola@uks.eu

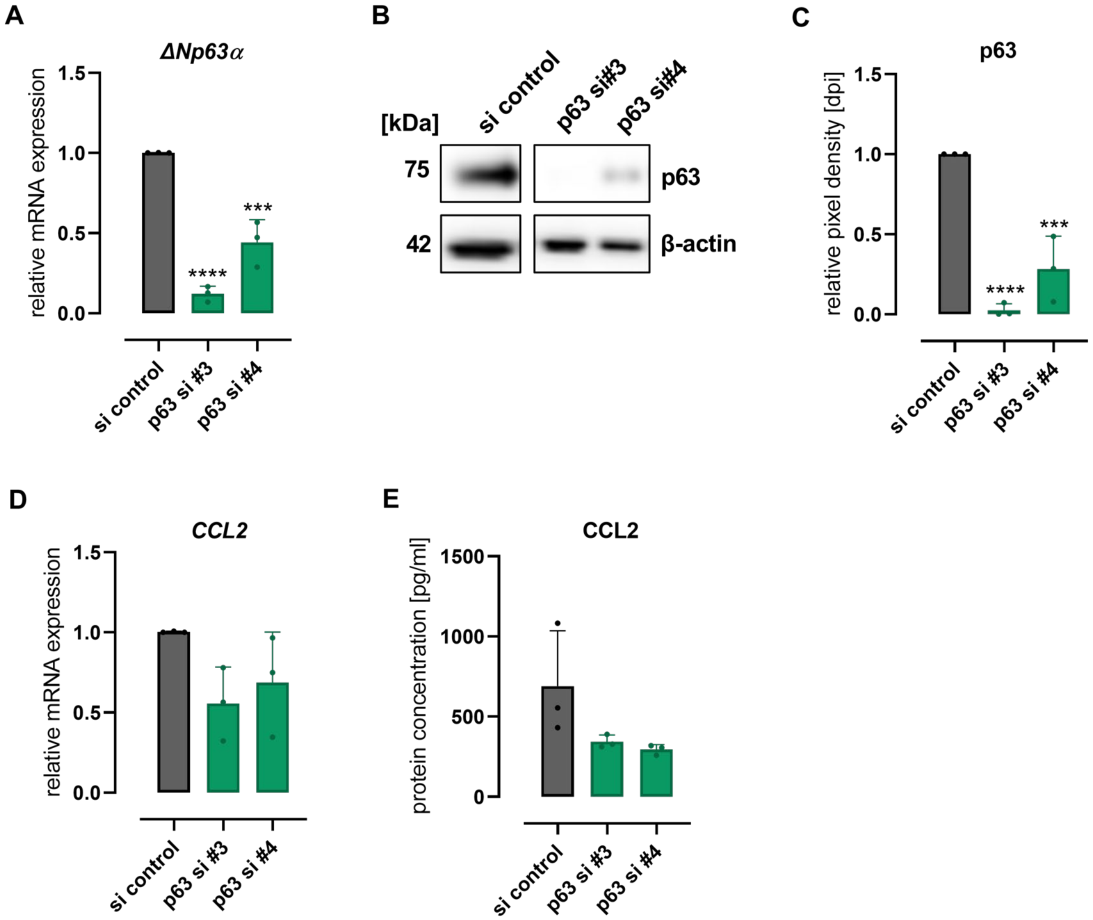

Supplementary Figure 2: p63 regulates CCL2 in HaCaT: A-E) p63 and CCL2 expression of HaCaT cells after treatment with siRNA targeting p63 or control siRNA (si). Expression of ΔNp63α was detected by qRT-PCR (A) and total p63 protein by immunoblot (representative image in B and densitometry of three independent experiments in C). CCL2 expression was quantified by qRT-PCR (D) and ELISA (E) of respective conditioned media. Results demonstrated as "relative mRNA expression" in A, D were normalized to RPL13a housekeeping gene expression, as "relative pixel density [dpi]" normalized to β-actin calculated by imageJ in C and as "protein concentration [pg/ml]" in E. Significant difference were calculated with one-way Anova and Tukey correction for multiple comparison and displayed as * $p < 0.05$, *** $p < 0.001$ and **** $p < 0.0001$.
