# Supplementary material for: The cutaneous beta human papillomavirus type 8 E6 protein induces CCL2 through the CEBPα/miR-203/p63 pathway to support an inflammatory microenvironment in epidermodysplasia verruciformis skin lesions
Source: Front Cell Infect Microbiol. 2024 Mar 6;14:1336492. doi: 10.3389/fcimb.2024.1336492 (PMC10953690; doi:10.3389/fcimb.2024.1336492)
Supplement: Supplementary file 3 [file Image_3.pdf]

The cutaneous beta human papillomavirus type 8 E6 protein induces CCL2 through the CEBPα/miR-203/p63 pathway to support an inflammatory microenvironment in epidermodysplasia verruciformis skin lesions

Luca Vella[1], Anna Sternjakob[1], Stefan Lohse[1], Alina Fingerle[1], Tanya Sperling[2], Claudia Wickenhauser[3], Michael Stöckle[4], Thomas Vogt[5], Klaus Roemer[6], Monika Ołdak[1,7], Sigrun Smola[1,8]*

[1]Institute of Virology, Saarland University Medical Center, Homburg/Saar, Germany

[2]Institute of Virology, University of Cologne, Cologne, Germany

[3]Institute of Pathology, University of Cologne, Cologne, Germany

[4]Department of Urology and Pediatric Urology, Saarland University Medical Center, Homburg/Saar, Germany

[5]Department of Dermatology, Saarland University Medical Center, Homburg/Saar, Germany

[6]Jose Carreras Center for Immune and Gene therapy, Saarland University Medical Center, Homburg/Saar, Germany

[7]Department of Histology and Embryology, Medical University of Warsaw, Warsaw, Poland

[8]Helmholtz Institute for Pharmaceutical Research Saarland (HIPS), Helmholtz Centre for Infection Research, Saarbrücken, Germany

*** Correspondence:**
Sigrun Smola
sigrun.smola@uks.eu

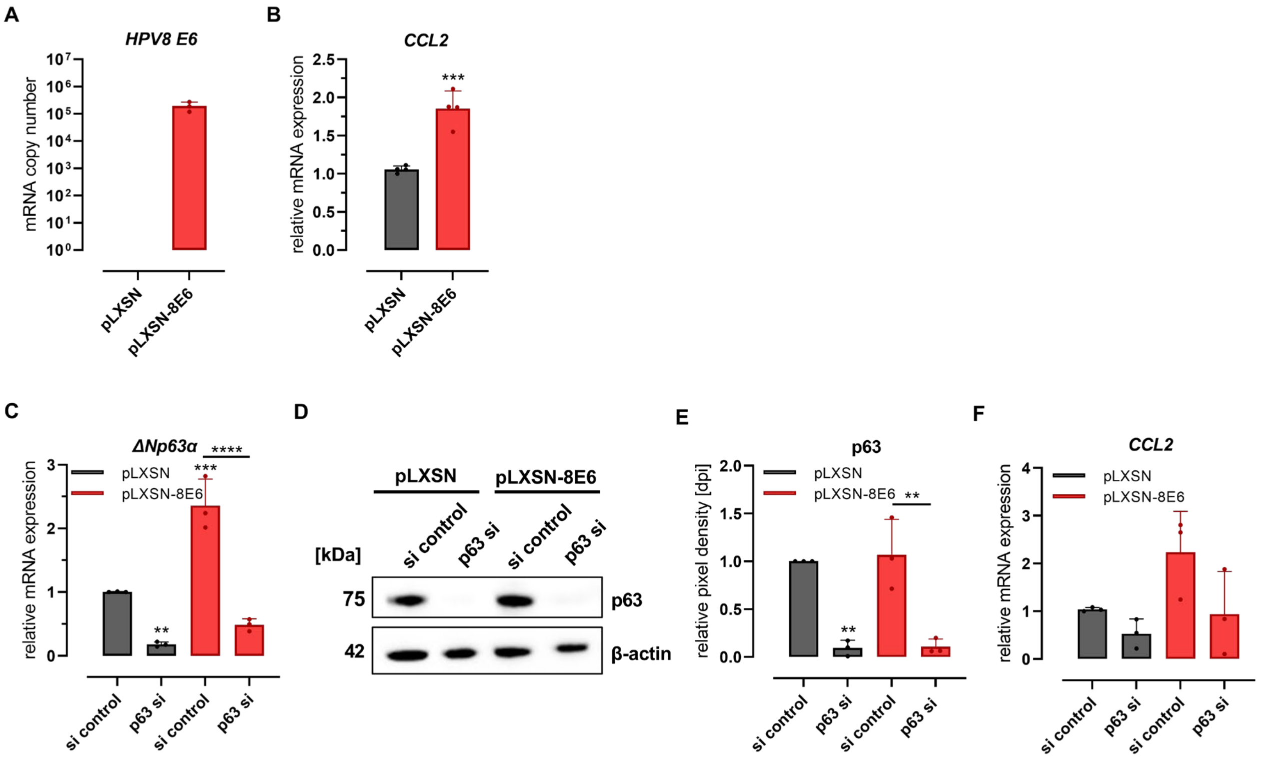

Figure S3: HPV8 E6 mediates CCL2 induction in NHK: (A-B) NHK were retrovirally engineered to express HPV8 E6 (red) or empty pLXSN vector (gray). Expression of HPV8 E6 (A) and CCL2 (B) mRNAs was determined by qRT-PCR. (C-F) NHK were treated with siRNA targeting p63 or control siRNA (si). Expression of ΔNp63α mRNA was detected by qRT-PCR (C), of total p63 protein by immunoblot (D, representative images, E, densitometry of three independent experiments), and of CCL2 mRNA by qRT-PCR (F). Results of qRT-PCR are demonstrated as "relative mRNA expression" normalized to RPL13a housekeeping gene expression and as "relative pixel density [dpi]" normalized to β-actin calculated by imageJ. Significant difference were calculated with two-tailed unpaired t-test (A-B) and two-way Anova and Sidak correction (C-F) for multiple comparison and displayed as * $p < 0.05$, ** $p < 0.01$, *** $p < 0.001$ and **** $p < 0.0001$.
