# Supplementary material for: The cutaneous beta human papillomavirus type 8 E6 protein induces CCL2 through the CEBPα/miR-203/p63 pathway to support an inflammatory microenvironment in epidermodysplasia verruciformis skin lesions
Source: Front Cell Infect Microbiol. 2024 Mar 6;14:1336492. doi: 10.3389/fcimb.2024.1336492 (PMC10953690; doi:10.3389/fcimb.2024.1336492)
Supplement: Supplementary file 4 [file Table_1.docx]

The cutaneous beta human papillomavirus type 8 E6 protein induces CCL2 through the CEBPα/miR-203/p63 pathway to support an inflammatory microenvironment in epidermodysplasia verruciformis skin lesions

**Luca Vella^1^, Anna Sternjakob^1^, Stefan Lohse^1^, Alina Fingerle^1^, Tanya Sperling^2^, Claudia Wickenhauser^3^, Michael Stöckle^4^, Thomas Vogt^5^, Klaus Roemer^6^, Monika Ołdak^1,7^, Sigrun Smola^1,8*^**

^1^Institute of Virology, Saarland University Medical Center, Homburg/Saar, Germany

^2^Institute of Virology, University of Cologne, Cologne, Germany

^3^Institute of Pathology, University of Cologne, Cologne, Germany

^4^Department of Urology and Pediatric Urology, Saarland University Medical Center, Homburg/Saar, Germany

^5^Department of Dermatology, Saarland University Medical Center, Homburg/Saar, Germany

^6^Jose Carreras Center for Immune and Gene therapy, Saarland University Medical Center, Homburg/Saar, Germany

^7^Department of Histology and Embryology, Medical University of Warsaw, Warsaw, Poland

^8^Helmholtz Institute for Pharmaceutical Research Saarland (HIPS), Helmholtz Centre for Infection Research, Saarbrücken, Germany

*** Correspondence:**Sigrun Smola
sigrun.smola@uks.eu

**Supplementary Table 1: Material identifiers**

| **Cell culture, medium, supplements, chemicals** | | | |
| --- | --- | --- | --- |
| HaCaT | ACC-771 | DSMZ | RRID:CVCL_0038 |
| KGM2 | C-39016 | PromoCell |  |
| DMEM | P04-01550 | Panbiotech |  |
| FCS |  | Sigma-Aldrich |  |
| Sodium pyruvate | S8636 | Sigma-Aldrich |  |
| Penicillin/Streptomycin | P4333 | Sigma-Aldrich |  |
| RPMI | P04-18047 | Panbiotech |  |
| G-418 | P06-16200 | Panbiotech |  |
| PBS | P04-37500 | Panbiotech |  |
| DMEM/F12 | P04-41500 | Panbiotech |  |
| PFA | 158127 | Sigma-Aldrich |  |
| DMSO | 67-68-5 | Sigma-Aldrich |  |
| Cristal violet | 115940 | Sigma-Aldrich |  |
| RS102895 | 2089 | Tocris Bioscience |  |
| **siRNA transfection, Reverse Transcription** | | | |
| CEBPα siRNA | L-006422 | Dharmacon |  |
| p63 siRNA | L-003330-00 | Dharmacon |  |
| Lipofectamin RNAiMAX | 13778075 | Fisher Scientific |  |
| Lipofectamin LTX | 15338100 | Fisher Scientific |  |
| Nucleospin RNA Kit | 740955 | Machery&Nagel |  |
| Maxima reverse transcriptase | EP0742 | Fisher Scientific |  |
| **Monocyte isolation** | | | |
| Pancoll | P04-60500 | Panbiotech |  |
| Transwell, 5 µm | 3421 | Corning |  |
| CD14 Microbeads | 130-050-201 | Miltenyi |  |
| Midi MACS separator | 130-042-301 | Miltenyi |  |
| **Antibodies, reagents, cytokines, ELISA, multiplex assays** | | | |
| p63 Ab | 39692 | Cell Signaling Technology | RRID:AB_2799159 |
| CEBPα Ab | 8178 | Cell Signaling Technology | RRID:AB_11178517 |
| ß-Actin Ab | A1978 | Sigma-Aldrich | RRID:AB_476692 |
| 7-AAD | 420403 | Biolegend |  |
| CCL2 Ab IHC | ab9669 | abcam | RRID:AB_2071551 |
| CCL2 Ab IF | 28D | Novus Biologicals |  |
| goat anti-rabbit POX | 12-348 | Sigma-Aldrich | RRID:AB_390191 |
| rabbit anti-mouse POX | AP160 | Sigma-Aldrich | RRID:AB_92529 |
| anti-human CD14-FITC | 555397 | BD Bioscience | RRID:AB_395798 |
| rhCCL2 | 300-04 | Peprotech |  |
| TNF-α | * | Boehringer Ingelheim |  |
| ELISA CCL2 Kit | 438804 | Biolegend |  |
| Cytokine Screening panel 48-plex | 12007283 | Biorad |  |
| RIPA buffer | R0278 | Sigma-Aldrich |  |
| Western Bright ECL | K-12049 | Biozym |  |
| Nitrocellulose membrane | 11979307 | Fisher Scientific |  |

* Recombinant human TNF-α was a gift from Dr. G. Adolf (Bender and Co/Boehringer Ingelheim Austria GmbH, Wien, Austria) [Hess, 1998 #545].

**Supplementary Table 2: Sequences of siRNAs**

| **Target** | **siRNA sequence** |
| --- | --- |
| Non-targeting siRNA control | 5‘-UGGUUUACAUGUUGUGUGA-3‘ |
| p63 siRNA  ON-TARGETplus SMARTpool siRNA TP73L (p63) | 5‘-GAUGAACUGUUAUACUUAC-3‘  5‘-CGACAGUCUUGUACAAUUU-3‘  5‘-GCACACAGACAAAUGAAUU-3‘  5‘-UCUAUCAGAUUGAGCAUUA-3  (Underlined sequences are single siRNA#3 und #4) |
| C/EBPα siRNA  ON-TARGET plus human CEBPα siRNA SMART pool | 5‘-ACAAUGACCGCCUGCGCAA-3‘  5‘-CACGAGACGUCCAUCGACA-3‘  5‘-GAACAGCUGAGCCGCGAAC-3‘  5‘-GAACAGCAACGAGUACCGG-3‘ |

**Supplementary Table 3: qRT-PCR primer (sense and antisense) and probes (Roche)**

| **Gene** | **sense** | **antisense** | **UPL probe (Roche)** |
| --- | --- | --- | --- |
| CCL2 | 5‘-AGTCTCTGCCGCCCTTCT-3‘ | 5‘-GTGACTGGGGCATTGATTG-3‘ | 40 |
| C/EBPα | 5‘-GTGGACAAGAACAGCAACGA-3‘ | 5‘-CACTGGTCAGCTCCAGCAC-3‘ | 84 |
| HPV8 E6 | 5‘-CCGCAACGTTTGAATTTAATG-3‘ | 5‘-ATTGAACGTCCTGTAGCTAATTCA-3‘ | 13 |
| HPV8 E7 | 5‘-AGGAATTACCAAACGAACAGGA-3‘ | 5‘-CACGGTGCAACAATTTTGAATA-3‘ | 63 |
| RPL13a | 5‘-AGCGGATGAACACCAACC-3‘ | 5‘-TTTGTGGGGCAGCATACTC-3‘ | 28 |
| ΔNp63α | 5‘-GGAAAACAATGCCCAGACTC-3‘ | 5‘-CTGCTGGTCCATGCTGTTC-3 | 45 |

**Supplementary Table 4: Cytokines detected by 48-plex immunoassay**

| CTACK (CCL27) | IL-18 | MCP-3 (CCL7) |
| --- | --- | --- |
| Eotaxin | IL1-RA | M-CSF |
| FGF basic | IL-1α | MIF |
| G-CSF | IL-1β | MIG (CXCL9) |
| GM-CSF | IL-2 | MIP-1α (CCL3) |
| GROα (CXCL1) | IL-2Rα | MIP-1β (CCL4) |
| HGF | IL-3 | PDGF-BB |
| IFN-α2 | IL-4 | RANTES (CCL5) |
| IFN-γ | IL-5 | SCF |
| IL-10 | IL-6 | SCGF-β |
| IL-12 (p40) | IL-7 | SDF-1α (CXCL12) |
| IL-12 (p70) | IL-8 | TNF-α |
| IL-13 | IL-9 | TNF-β |
| IL-15 | IP-10 (CXCL10) | TRAIL |
| IL-16 | LIF | VEGF |
| IL-17A | MCP-1 (CCL2) | β-NGF |
