# Supplementary material for: The cutaneous beta human papillomavirus type 8 E6 protein induces CCL2 through the CEBPα/miR-203/p63 pathway to support an inflammatory microenvironment in epidermodysplasia verruciformis skin lesions
Source: Front Cell Infect Microbiol. 2024 Mar 6;14:1336492. doi: 10.3389/fcimb.2024.1336492 (PMC10953690; doi:10.3389/fcimb.2024.1336492)
Supplement: Supplementary file 5 [file Table_2.docx]

The cutaneous beta human papillomavirus type 8 E6 protein induces CCL2 through the CEBPα/miR-203/p63 pathway to support an inflammatory microenvironment in epidermodysplasia verruciformis skin lesions

**Luca Vella^1^, Anna Sternjakob^1^, Stefan Lohse^1^, Alina Fingerle^1^, Tanya Sperling^2^, Claudia Wickenhauser^3^, Michael Stöckle^4^, Thomas Vogt^5^, Klaus Roemer^6^, Monika Ołdak^1,7^, Sigrun Smola^1,8*^**

^1^Institute of Virology, Saarland University Medical Center, Homburg/Saar, Germany

^2^Institute of Virology, University of Cologne, Cologne, Germany

^3^Institute of Pathology, University of Cologne, Cologne, Germany

^4^Department of Urology and Pediatric Urology, Saarland University Medical Center, Homburg/Saar, Germany

^5^Department of Dermatology, Saarland University Medical Center, Homburg/Saar, Germany

^6^Jose Carreras Center for Immune and Gene therapy, Saarland University Medical Center, Homburg/Saar, Germany

^7^Department of Histology and Embryology, Medical University of Warsaw, Warsaw, Poland

^8^Helmholtz Institute for Pharmaceutical Research Saarland (HIPS), Helmholtz Centre for Infection Research, Saarbrücken, Germany

*** Correspondence:**Sigrun Smola
sigrun.smola@uks.eu

**Supplementary Table 2: Sequences of siRNAs**

| **Target** | **siRNA sequence** |
| --- | --- |
| Non-targeting siRNA control | 5‘-UGGUUUACAUGUUGUGUGA-3‘ |
| p63 siRNA  ON-TARGETplus SMARTpool siRNA TP73L (p63) | 5‘-GAUGAACUGUUAUACUUAC-3‘  5‘-CGACAGUCUUGUACAAUUU-3‘  5‘-GCACACAGACAAAUGAAUU-3‘  5‘-UCUAUCAGAUUGAGCAUUA-3  (Underlined sequences are single siRNA#3 und #4) |
| C/EBPα siRNA  ON-TARGET plus human CEBPα siRNA SMART pool | 5‘-ACAAUGACCGCCUGCGCAA-3‘  5‘-CACGAGACGUCCAUCGACA-3‘  5‘-GAACAGCUGAGCCGCGAAC-3‘  5‘-GAACAGCAACGAGUACCGG-3‘ |
