# Supplementary material for: The cutaneous beta human papillomavirus type 8 E6 protein induces CCL2 through the CEBPα/miR-203/p63 pathway to support an inflammatory microenvironment in epidermodysplasia verruciformis skin lesions
Source: Front Cell Infect Microbiol. 2024 Mar 6;14:1336492. doi: 10.3389/fcimb.2024.1336492 (PMC10953690; doi:10.3389/fcimb.2024.1336492)
Supplement: Supplementary file 6 [file Table_3.docx]

The cutaneous beta human papillomavirus type 8 E6 protein induces CCL2 through the CEBPα/miR-203/p63 pathway to support an inflammatory microenvironment in epidermodysplasia verruciformis skin lesions

**Luca Vella^1^, Anna Sternjakob^1^, Stefan Lohse^1^, Alina Fingerle^1^, Tanya Sperling^2^, Claudia Wickenhauser^3^, Michael Stöckle^4^, Thomas Vogt^5^, Klaus Roemer^6^, Monika Ołdak^1,7^, Sigrun Smola^1,8*^**

^1^Institute of Virology, Saarland University Medical Center, Homburg/Saar, Germany

^2^Institute of Virology, University of Cologne, Cologne, Germany

^3^Institute of Pathology, University of Cologne, Cologne, Germany

^4^Department of Urology and Pediatric Urology, Saarland University Medical Center, Homburg/Saar, Germany

^5^Department of Dermatology, Saarland University Medical Center, Homburg/Saar, Germany

^6^Jose Carreras Center for Immune and Gene therapy, Saarland University Medical Center, Homburg/Saar, Germany

^7^Department of Histology and Embryology, Medical University of Warsaw, Warsaw, Poland

^8^Helmholtz Institute for Pharmaceutical Research Saarland (HIPS), Helmholtz Centre for Infection Research, Saarbrücken, Germany

*** Correspondence:**Sigrun Smola
sigrun.smola@uks.eu

**Supplementary Table 3: qRT-PCR primer (sense and antisense) and probes (Roche)**

| **Gene** | **sense** | **antisense** | **UPL probe (Roche)** |
| --- | --- | --- | --- |
| CCL2 | 5‘-AGTCTCTGCCGCCCTTCT-3‘ | 5‘-GTGACTGGGGCATTGATTG-3‘ | 40 |
| C/EBPα | 5‘-GTGGACAAGAACAGCAACGA-3‘ | 5‘-CACTGGTCAGCTCCAGCAC-3‘ | 84 |
| HPV8 E6 | 5‘-CCGCAACGTTTGAATTTAATG-3‘ | 5‘-ATTGAACGTCCTGTAGCTAATTCA-3‘ | 13 |
| HPV8 E7 | 5‘-AGGAATTACCAAACGAACAGGA-3‘ | 5‘-CACGGTGCAACAATTTTGAATA-3‘ | 63 |
| RPL13a | 5‘-AGCGGATGAACACCAACC-3‘ | 5‘-TTTGTGGGGCAGCATACTC-3‘ | 28 |
| ΔNp63α | 5‘-GGAAAACAATGCCCAGACTC-3‘ | 5‘-CTGCTGGTCCATGCTGTTC-3 | 45 |
