# Supplementary material for: The cutaneous beta human papillomavirus type 8 E6 protein induces CCL2 through the CEBPα/miR-203/p63 pathway to support an inflammatory microenvironment in epidermodysplasia verruciformis skin lesions
Source: Front Cell Infect Microbiol. 2024 Mar 6;14:1336492. doi: 10.3389/fcimb.2024.1336492 (PMC10953690; doi:10.3389/fcimb.2024.1336492)
Supplement: Supplementary file 7 [file Table_4.docx]

The cutaneous beta human papillomavirus type 8 E6 protein induces CCL2 through the CEBPα/miR-203/p63 pathway to support an inflammatory microenvironment in epidermodysplasia verruciformis skin lesions

**Luca Vella^1^, Anna Sternjakob^1^, Stefan Lohse^1^, Alina Fingerle^1^, Tanya Sperling^2^, Claudia Wickenhauser^3^, Michael Stöckle^4^, Thomas Vogt^5^, Klaus Roemer^6^, Monika Ołdak^1,7^, Sigrun Smola^1,8*^**

^1^Institute of Virology, Saarland University Medical Center, Homburg/Saar, Germany

^2^Institute of Virology, University of Cologne, Cologne, Germany

^3^Institute of Pathology, University of Cologne, Cologne, Germany

^4^Department of Urology and Pediatric Urology, Saarland University Medical Center, Homburg/Saar, Germany

^5^Department of Dermatology, Saarland University Medical Center, Homburg/Saar, Germany

^6^Jose Carreras Center for Immune and Gene therapy, Saarland University Medical Center, Homburg/Saar, Germany

^7^Department of Histology and Embryology, Medical University of Warsaw, Warsaw, Poland

^8^Helmholtz Institute for Pharmaceutical Research Saarland (HIPS), Helmholtz Centre for Infection Research, Saarbrücken, Germany

*** Correspondence:**Sigrun Smola
sigrun.smola@uks.eu

**Supplementary Table 4: Cytokines detected by 48-plex immunoassay**

| CTACK (CCL27) | IL-18 | MCP-3 (CCL7) |
| --- | --- | --- |
| Eotaxin | IL1-RA | M-CSF |
| FGF basic | IL-1α | MIF |
| G-CSF | IL-1β | MIG (CXCL9) |
| GM-CSF | IL-2 | MIP-1α (CCL3) |
| GROα (CXCL1) | IL-2Rα | MIP-1β (CCL4) |
| HGF | IL-3 | PDGF-BB |
| IFN-α2 | IL-4 | RANTES (CCL5) |
| IFN-γ | IL-5 | SCF |
| IL-10 | IL-6 | SCGF-β |
| IL-12 (p40) | IL-7 | SDF-1α (CXCL12) |
| IL-12 (p70) | IL-8 | TNF-α |
| IL-13 | IL-9 | TNF-β |
| IL-15 | IP-10 (CXCL10) | TRAIL |
| IL-16 | LIF | VEGF |
| IL-17A | MCP-1 (CCL2) | β-NGF |
